# Targeting survivin with Tanshinone IIA inhibits tumor growth and overcomes chemoresistance in colorectal cancer

**DOI:** 10.1038/s41420-023-01622-8

**Published:** 2023-09-25

**Authors:** Yaoquan Cao, Haibo Tang, Guohui Wang, Pengzhou Li, Zhi Song, Weizheng Li, Xulong Sun, Xiaoxiao Zhong, Qianqian Yu, Shaihong Zhu, Liyong Zhu

**Affiliations:** Department of General Surgery, Third Xiangya Hospital, Central South University, Changsha, 410013 China

**Keywords:** Cancer therapeutic resistance, Drug discovery and development

## Abstract

The inhibitor of apoptosis protein survivin has a critical regulatory role in carcinogenesis and treatment tolerance in colorectal cancer (CRC). However, the targeted drugs for survivin protein are extremely limited. In the present research, we discovered that Tanshinone IIA (Tan IIA) played a dual regulatory role in inhibiting tumorigenesis and reversing 5-Fu tolerance via modulating the expression and phosphorylation of survivin in CRC cells. Mechanistically, Tan IIA suppressed the Akt/WEE1/CDK1 signaling pathway, which led to the downregulation of survivin Thr34 phosphorylation and destruction of the interaction between USP1 and survivin to promote survivin ubiquitination and degradation. Furthermore, Tan IIA significantly facilitated chemoresistant CRC cells to 5-Fu sensitivity. These results revealed that Tan IIA possessed a strong antitumor activity against CRC cells and could act as an up-and-coming agent for treating CRC and overcoming chemotherapy resistance.

## Introduction

Colorectal cancer (CRC) is one of the most common gastrointestinal malignancies and the second-leading cause of cancer-related deaths worldwide, with 3.2 million CRC cases and 1.6 million deaths expected by 2040 [1]. The pathogenesis of CRC is multifactorial etiology, involving genetic factors, excess body weight, physical inactivity, unhealthy lifestyle (excessive intake of red and processed meat, insufficient intake of dietary fiber, high alcohol consumption, and smoking) and advancing age [2, 3]. In recent decades, the prognosis of patients with CRC has improved due to advancements in perioperative management, neoadjuvant therapy, and adjuvant therapy [4]. Chemotherapy, one of the major traditional therapies, is indispensable in treating advanced CRC patients. However, the patients eventually experience tumor progression because of acquired chemoresistance, and the 5-year survival rate of advanced CRC is lower than 10% [5, 6]. Therefore, to further elucidating the molecular mechanisms of chemoresistance in CRC and finding solutions to confer CRC cells re-sensitive to chemotherapy are of paramount importance.

Apoptosis, a tightly regulated cell death program, performs crucial functions to limit cell population and maintain tissue homeostasis [7]. Aberrant apoptosis is widely regarded as the acquired characteristic of cancer cells, involving carcinogenesis, tumor progression, and tolerance of antitumor treatment [8, 9]. Survivin is the smallest member of the inhibitor of apoptosis (IAP) family and plays a critical role in regulating the cell division process and programmed cell death. Survivin serves its regulatory function in cell cycle progression and mitosis via its role in the chromosomal passenger complex to regulate chromosome segregation and cytokinesis. It exerts its antiapoptosis effect via interaction with X-linked IAP (XIAP) or hepatitis B X-interacting protein to inhibit apoptosis activation [10, 11]. Survivin is broadly expressed in embryonic and fetal organs but is rarely detected in normal adult tissues [12, 13]. Moreover, numerous studies have revealed that the expression of survivin is highly upregulated in most human malignancies, such as lung cancer [14], breast cancer [15], colorectal cancer [16] and glioblastoma multiforme [17], which enables cancer cells to increase proliferation, avoid apoptosis, tumor recurrence, metastasis, and chemotherapy/radiotherapy resistance [18, 19]. Therefore, increasing attention has been paid to discovering inhibitors targeting survivin to promote cancer therapeutic strategies.

Recently, natural products extracted from plants have attracted great attention due to their excellent biological activities against bacteria, inflammation, viruses, and tumors, as well as good tolerance and less toxicity [20, 21]. Tanshinone IIA (Tan IIA) is the main lipophilic active component of the dried root and rhizome of *Salvia miltiorrhiza*, a traditional Chinese medicinal herb widely used in the clinic [22, 23]. Tan IIA has attracted considerable attention from researchers due to its possession of various pharmacological activities, including antioxidant, neuron-protective, anti-inflammatory, antifatigue, antitumor, etc. [24–26]. Li et al. revealed that Tan IIA restrained pulmonary fibrosis by regulating the Keap1/Nrf2 signal pathway to activate Sestrin2 [27]. Another study demonstrated that Tan IIA protected against doxorubicin-induced cardiotoxicity and was a novel agent for heart failure treatment through activating the DAXX/MEK/ERK1/2 signal pathway [28]. For its antitumor activity, Tan IIA was found to inhibit the growth of triple-negative breast cancer(TNBC) cells by upregulating TP53 and decreasing Bcl-1 expression [29]. Although some previous studies have reported that Tan IIA has extensive antitumor effects on several human tumor cell lines, the research on the mechanism of Tan IIA is relatively scattered, and its biological effect on colorectal cancer cells is rarely investigated. The present study focused on the bioactivity of Tan IIA on CRC cells clarified that Tan IIA exerted strong antitumor activity against CRC cells both in vitro and in vivo, and investigated the underlying mechanism of this pharmacological function.

## Results

### Tan IIA suppresses proliferation and activates mitochondrial apoptosis signals

**Fig. 1:**
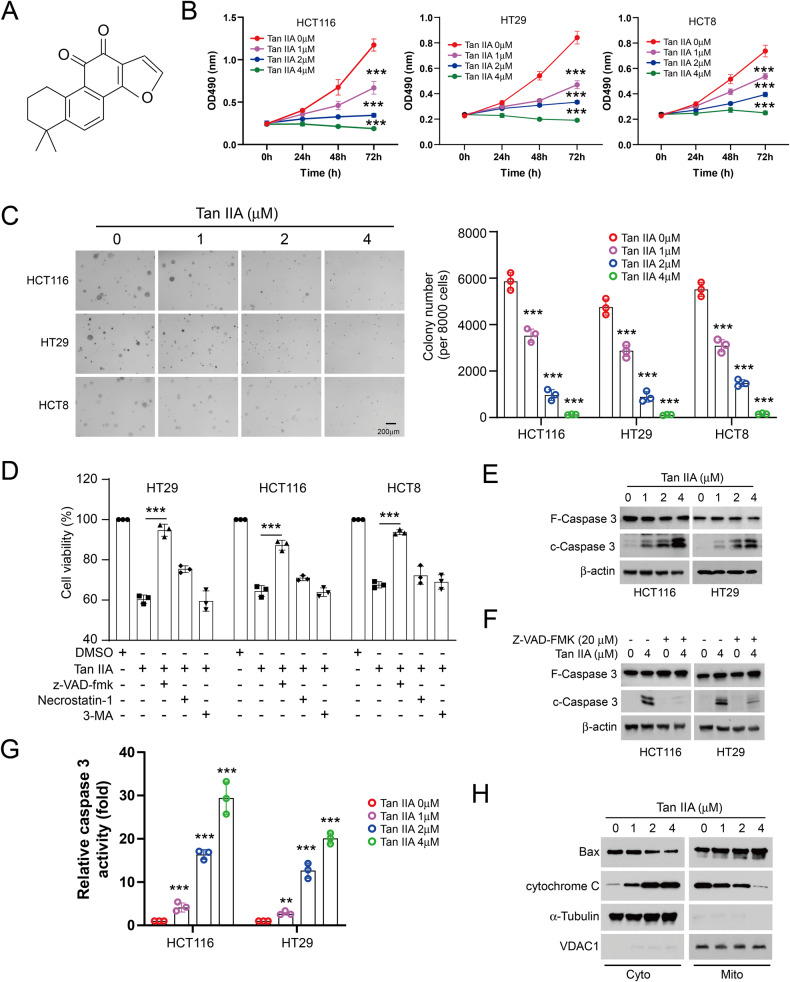
Tanshinone IIA (Tan IIA) suppresses CRC cells. **A** The chemical structure of Tan IIA. **B** The effect of Tan IIA on cell viability of CRC cells. CRC cell lines HCT116, HT29 and HCT8 were incubated with DMSO or Tan IIA for 24, 48 or 72 h. Cell viability was assayed by MTS analysis. **C** The effect of Tan IIA on colony formation of CRC cell lines HCT116, HT29 and HCT8 that were treated with DMSO control or Tan IIA. A soft agar assay was used to measure the colony-forming activity of CRC cells. **D** z-VAD-fmk reversed Tan IIA-reduced cell viability. HT29, HCT116 and HCT8 cells were pretreated with 3-MA, Necrostatin-1, or z-VAD-fmk for 4 h, followed by DMSO control or Tan IIA (4 μM) treatment for 24 h. **E** HCT116 and HT29 cells were treated with Tan IIA for 24 h. The expression of cleaved-caspase 3 in whole-cell extract (WCE) was determined through IB analysis. **F** HCT116 and HT29 cells were pretreated with z-VAD-fmk for 4 h, followed by Tan IIA treatment for 24 h, and IB analysis was performed on WCE. **G** HCT116 and HT29 cells were treated with Tan IIA for 24 h. Caspase-3 activity was evaluated by caspase-3 activity assay. **H** CRC cells were treated with Tan IIA for 24 h. Isolation and analysis of subcellular fragments by IB. Cyto, cytosolic fraction, Mito, mitochondrial fraction. ***P < 0.001.

Tan IIA (Fig. 1A) exhibits the antitumor effect on human cancer cells [30]. However, the effect of Tan IIA on CRC cells remained elusive. The MTS assay showed that the viability of HCT116, HT29, and HCT8 cells was inhibited by Tan IIA time- and dose-dependently (Fig. 1B). Moreover, the colony formation of CRC cells in soft agar was diminished obviously by Tan IIA, and 4 μM Tan IIA reduced colony formation by over 90% (Fig. 1C). Pretreatment with z-VAD-fmk, but not Nec-1 or 3-MA, reversed Tan IIA-reduced cell viability, indicating that Tan IIA activating intrinsic apoptosis signaling in CRC cells (Fig. 1D). Likewise, the immunoblotting (IB) results confirmed that Tan IIA increased the protein levels of cleaved-caspase 3 in HCT116 and HT29 cells (Fig. 1E), which was impaired by z-VAD-fmk (Fig. 1F). Furthermore, Tan IIA enhanced caspase 3 activity dose-dependently (Fig. 1G). By isolating subcellular fractions of HT29 cells, we found that cytochrome C protein levels were raised in the cytoplasm fractions with Tan IIA treatment and reduced in the mitochondria dose-dependently (Fig. 1H). In addition, the Bax protein levels in the mitochondria were promoted followed by Tan IIA treatment (Fig. 1H). Our data suggested that Tan IIA inhibits cell viability of CRC cells and activates intrinsic apoptosis.

### Tan IIA downregulates survivin expression via promoting its ubiquitination

**Fig. 2:**
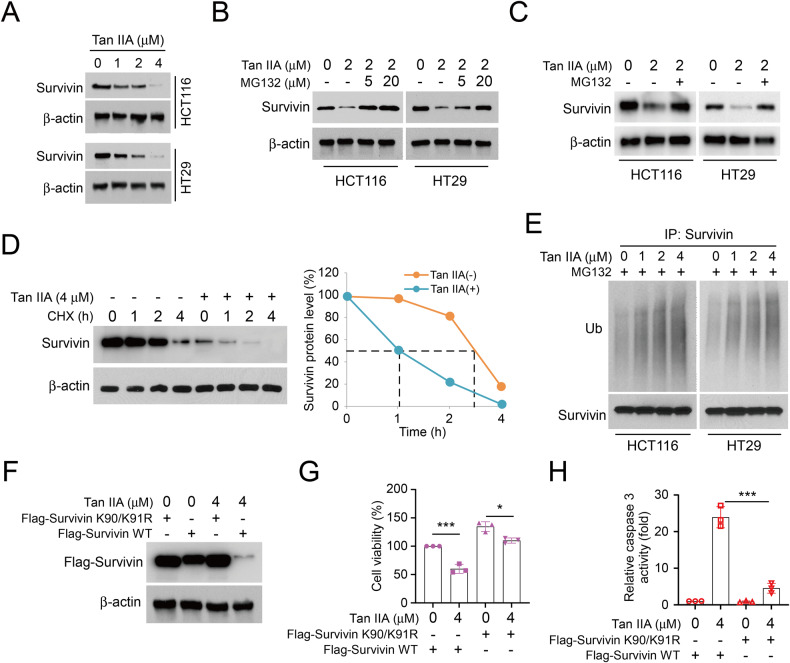
Tan IIA reduces survivin expression in CRC cells. **A** HCT116 and HT29 cells were treated with Tan IIA for 24 h, and WCE was subjected to IB analysis. **B** HCT116 and HT29 cells were treated with Tan IIA (2 µM) for 24 h and incubated with various concentrations of MG132 (20 µM) for 6 h. IB analysis was performed on WCE. **C** Tan IIA-induced survivin downregulation was rescued by MG132. HCT116 and HT29 cells were treated with Tan IIA (2 µM) and then with MG132 (20 µM) treatment for 6 h, IB analysis was performed on WCE. **D** Tan IIA shortened survivin protein half-life. CRC cells were treated with 4 μM Tan IIA or DMSO for 24 h, then incubated with cycloheximide (CHX) at different time points, and WCE was subjected to IB analysis. **E** Tan IIA stimulated survivin ubiquitination in a dose-dependent manner. HCT116 and HT29 cells were treated with Tan IIA for 24 h, WCE was subjected to ubiquitination analysis. CRC cells were transfected with Flag-Survivin-WT or K90/91 R mutant and then treated with Tan IIA (4 µM) for 24 h. IB assay (**F**), MTS assay (**G**) and cleaved-caspase-3 activity assay (**H**) were performed on cells. *P < 0.05, ***P < 0.001.

To determine whether Tan IIA-inhibited CRC cells depend on the downregulation of survivin, an IB assay was performed in CRC cells with Tan IIA treatment. The results suggested that Tan IIA decreased survivin protein level in HCT116 and HT29 cells dose-dependently (Fig. 2A). In addition, Tan IIA-induced survivin downregulation was restored by MG132, the proteasome inhibitor (Fig. 2B, C). Further analysis revealed that the half-life of survivin was shortened from 3 h to 1 h following Tan IIA treatment (Fig. 2D). The findings indicated that Tan IIA-induced survivin reduction was connected with survivin degradation. By performing ubiquitination analysis, we discovered that Tan IIA promoted survivin ubiquitination dose-dependently in CRC cell lines HCT116 and HT29 (Fig. 2E). Moreover, our immunoblotting results suggested that survivin expression of HCT116 cells transfected survivin wild-type, but not K90R/K91R double mutant (two ubiquitination sites required for survivin degradation) was markedly reduced following Tan IIA treatment (Fig. 2F). Additionally, Tan IIA treatment significantly decreased the cell viability and increased caspase 3 activity in CRC cells expressing survivin WT (Fig. 2G, H). These results suggested that Tan IIA downregulates survivin expression by promoting survivin ubiquitination and degradation.

### Tan IIA reduces survivin phosphorylation at Thr34 to promote apoptosis and survivin destruction

**Fig. 3:**
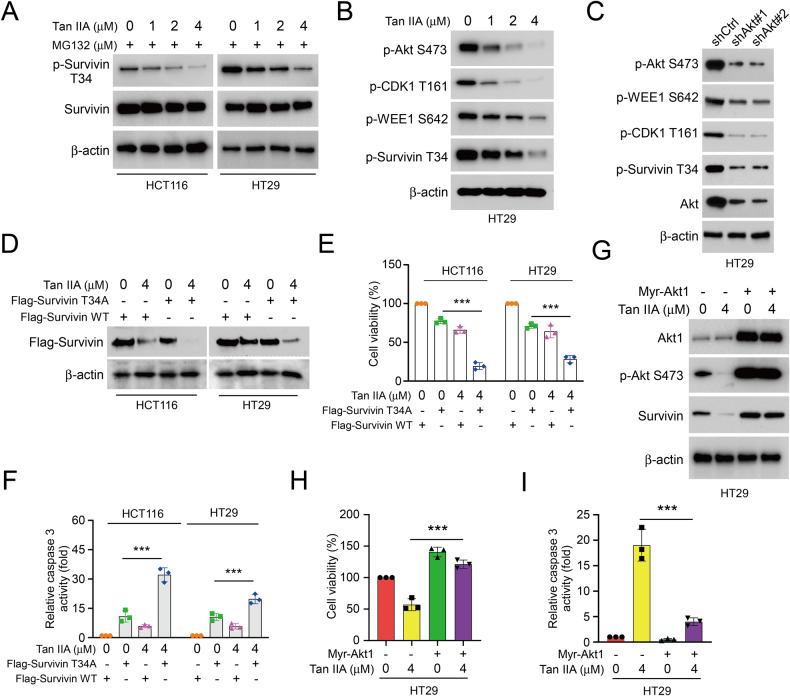
Reduction of survivin Thr34 phosphorylation caused by Tan IIA is required to ubiquitinate survivin in CRC cells. **A** CRC cells were treated with Tan IIA for 24 h, followed by MG132 treated for 6 h, and WCE was subjected to IB analysis. **B** CRC cells were treated with tanshinone IIA for 24 h, WCE was subjected to IB analysis. **C** HT29 cells were transfected with shCtrl or shAkt and then with Tan IIA treatment for 24 h, and WCE was subjected to IB analysis. HCT116 and HT29 cells were transfected with Flag-Survivin-WT or T34A and then with or without Tan IIA treatment (4 µM) for 24 h. IB assay (**D**), MTS assay (**E**) and cleaved-caspase-3 activity assay (**F**) were performed on cells. HT29 cells were transfected with Myr-Akt1 and then with Tan IIA treatment (4 µM) for 24 h, IB assay (**G**), MTS assay (**H**) and caspase-3 activity assay (**I**) were performed on cells. ***P < 0.001.

Phosphorylating survivin at Thr34 is crucial for the stability and biofunction of survivin. Thus, we examined whether Tan IIA impacted survivin phosphorylation. The immunoblotting results suggested that Tan IIA reduced survivin Thr34 phosphorylation dose-dependently (Fig. 3A). As shown in Fig. 3B, Tan IIA suppressed Akt signal pathways. The activation of Akt Ser473 and its downstream target kinases, CDK1 Thr161, WEE1 Ser642, and survivin Thr34 were suppressed coordinately after treatment with Tan IIA in HT29 cells (Fig. 3B). Similarly, following the knockdown of Akt via shRNA the phosphorylation of CDK1 (Thr161), WEE1 (Ser642) and survivin (Thr34) were suppressed in HT29 cells (Fig. 3C). Moreover, survivin WT or T34A mutant was used to overexpress in HCT116 and HT29 cells. We found that survivin T34A mutant protein levels were reduced more obviously following treatment with Tan IIA (Fig. 3D). Likewise, the cell viability (Fig. 3E) was decreased while relative caspase 3 activity (Fig. 3F) was increased in survivin T34A overexpressed HCT116 and HT29 cells. Furthermore, constitutively activated Akt (Myr-Akt1) was transfected into HT29 cells. We found that Akt ectopic overexpression reversed Tan IIA-induced reduction of survivin and Akt (S473) phosphorylation in HT29 cells (Fig. 3G). Consistently, Myr-Akt1 recovered cell viability and reduced relative caspase 3 activity in HT29 cells following Tan IIA treatment (Fig. 3H, I). These data indicated that Tan IIA inhibits survivin Thr34 phosphorylation and induces apoptosis by suppressing Akt signal pathways activation.

### Tan IIA inhibits the interaction between survivin and USP1

**Fig. 4:**
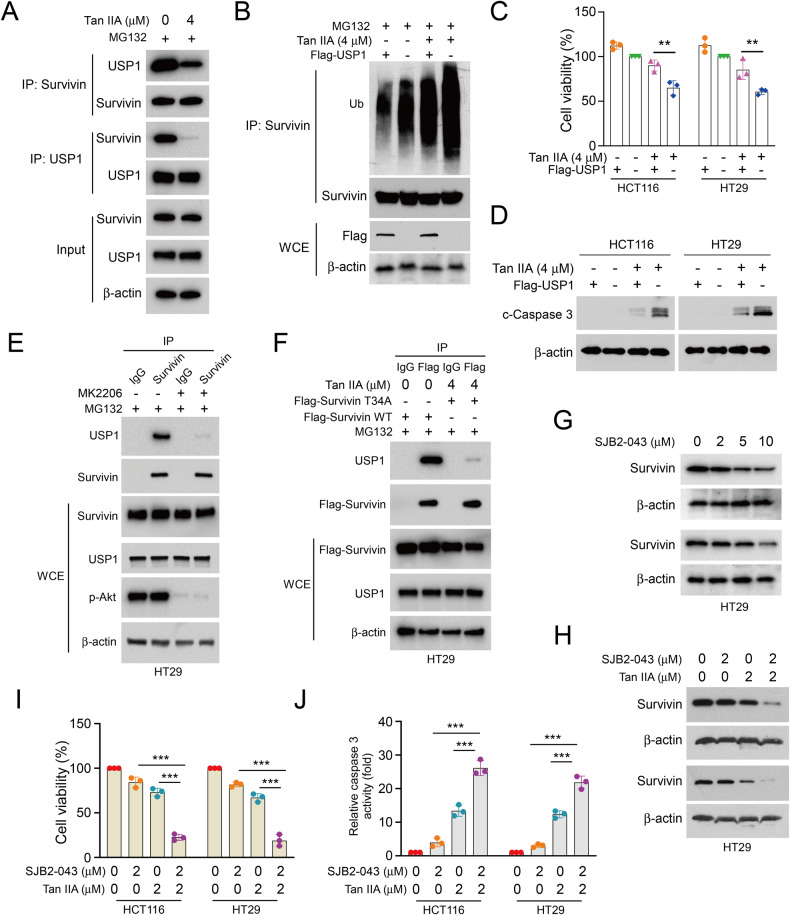
Tan IIA interferes with the binding of USP1 with Survivin. **A** HCT116 cells were pretreated with Tan IIA (4 µM) for 24 h and then incubated with MG132 (20 µM) for 6 h before IB analysis. **B**–**D** HCT116 and HT29 cells were transfected with Flag-USP1, followed by Tan IIA treatment (4 µM) for 24 h. **B** Ubiquitylation assay, (**C**) MTS analysis and (**D**) IB assay. **E** HT29 cells were treated with MK2206 (2 µM) and MG132, followed by co-IP and IB analysis. **F** HT29 cells were transfected with Flag-Survivin-WT or T34A mutant and then pretreated with Tan IIA (4 µM) for 24 h before co-IP and IB analysis. **G** HT29 cells were pretreated with various concentrations of SJB2-043 for 24 h before IB analysis. HT29 cells were pretreated with SJB2-043 (2 µM) and/or Tan IIA (2 µM) for 24 h, then subjected to IB assay (**H**), MTS assay (**I**) and caspase-3 activity assay (**J**). **P < 0.01, ***P < 0.001.

Recent research has shown that USP1 interacts with survivin and promotes survivin deubiquitination and stability [31]. Thus, we performed a co-IP assay to examine whether Tan IIA impacted the combination of USP1 with survivin. The IB results suggested that Tan IIA suppressed USP1 interacting with survivin in CRC cells (Fig. 4A). Moreover, USP1 inhibited survivin ubiquitination level, which was recovered in Tan IIA-treated CRC cells (Fig. 4B). Further research discovered that USP1 recovered the decrease of cell viability and reversed the increase of caspase 3 expressions in Tan IIA-treated HCT116 and HT29 cells (Fig. 4C, D). We next treated HT29 cells with MK2206 to inhibit Akt activation, finding that the inhibitor diminished the binding of USP1 with survivin (Fig. 4E). Furthermore, the IB data suggested that Tan IIA suppressed USP1 interacting with survivin Thr34 (Fig. 4F). We further treated HCT116 and HT29 cells with SJB2-043, a USP1 inhibitor, finding that survivin protein levels were decreased dose-dependently (Fig. 4G). As shown in Fig. 4H–J, The IB results suggested that combining SJB2-043 with Tan IIA reduced survivin protein levels more powerfully than alone treatment (Fig. 4H). Additionally, the cell viability was reduced, and relative caspase 3 activity was increased via combining SJB2-043 with Tan IIA more strongly than alone treatment (Fig. 4I, J). Taken together, the results suggested that Tan IIA suppresses the combination of USP1 with survivin to promote survivin ubiquitination, and combined USP1 inhibitor with Tan IIA plays a synergistic suppression role in CRC cells.

### Tan IIA suppresses tumor growth in vivo

**Fig. 5:**
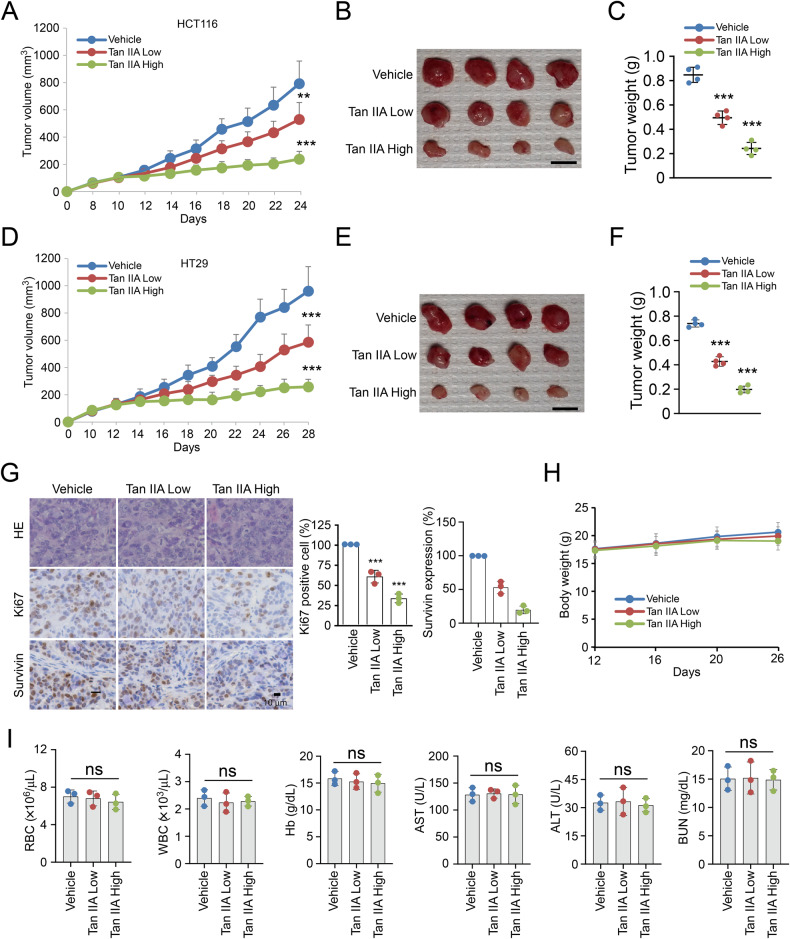
Tan IIA suppresses xenograft tumor growth in vivo. **A**–**F** HCT116- and HT29-derived xenograft tumor growth were suppressed by Tan IIA. The tumor volume (**A**), mass (**B**) and weight (**C**) of vehicle- and Tan IIA-treated HCT116 tumors. The tumor volume (**D**), mass (**E**) and weight (**F**) of vehicle- and Tan IIA-treated HT29 tumors. For (**B**) and (**E**), scale bar, 1 cm. **G** Representative images of Survivin and Ki-67 analyzed by IHC staining in HT29 tumors (left). Scale bar, 25 μm. Right, quantification of survivin and Ki-67. **H** The body weights of HT29 tumor-bearing mice with Tan IIA or vehicle therapy. **I** Mice blood assay following Tan IIA or vehicle treatment. **P < 0.01, ***P < 0.001.

The xenograft model was performed to examine the in vivo antitumor activity of Tan IIA. The data revealed that the development of HCT116- and HT29-derived xenograft tumors were suppressed obviously by Tan IIA (Fig. 5A–F). The volume (Fig. 5A, B) and weight (Fig. 5C) of HCT116 tumors were inhibited with Tan IIA treatment dose-dependently. Tan IIA exhibited a similar antitumor activity in the HT29 xenograft tumor model (Fig. 5D–F). Moreover, IHC results indicate that survivin and Ki-67 protein levels were reduced significantly by Tan IIA in HT29 xenograft tumors, especially with the higher dose of Tan IIA treatment (Fig. 5G). Furthermore, the body weight of Tan IIA-treated nude mice showed no significant change after Tan IIA treatment for 2 weeks (Fig. 5H). Likewise, blood analysis discovered that Tan IIA was unaffected significantly on RBC, WBC, Hb, AST, ALT, and BUN levels (Fig. 5I). The results indicate that Tan IIA with good tolerance can effectively suppress CRC tumor development in vivo.

### The 5-Fu resistance is dependent on survivin overexpression in CRC cells

**Fig. 6:**
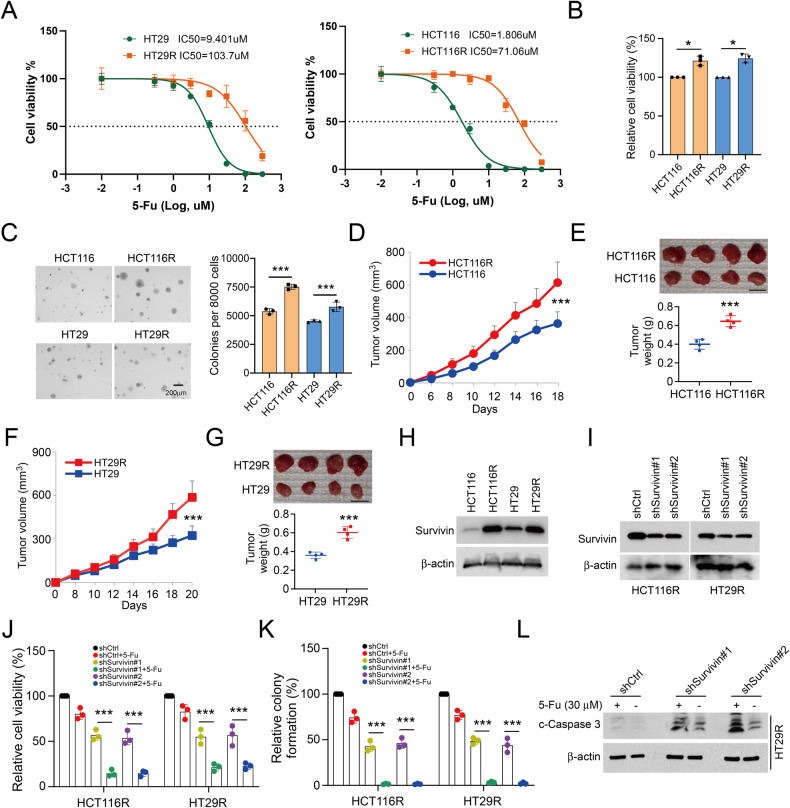
Survivin overexpressed in 5-Fu resistant CRC cells. **A** the IC50 of HT29/HT29R and HCT116/HCT116R cells with 5-Fu treatment. MTS and soft agar assays were used to detect cell viability (**B**) and colony formation (**C**) of HT29/HT29R and HCT116/HCT116R cells. **D**, **E** The in vivo tumorigenesis of HCT116 and HCT116R cells. **D** tumor volume; **E** tumor mass and weight. **F**, **G** The in vivo tumorigenesis of HT29 and HT29R cells. **F** tumor volume; **G** tumor mass and weight. **H** IB assay was used to detect survivin expression in HCT116/HCT116R and HT29/HT29R cells. **I** Knockdown of survivin in HCT116R and HT29R cells was measured by IB assay. Survivin knockdown HCT116R and HT29R cells were treated with 5-Fu (30 µM) or DMSO control and subjected to MTS (**J**) and soft agar (**K**) analysis. (**L**) IB assay of apoptosis in survivin knockdown HT29R with 5-Fu treatment. *P < 0.05, ***P < 0.001.

Extensive research has indicated that survivin is tightly related to chemoresistance. Two 5-Fu resistant CRC cells (HT29R/HCT116R) were established using the parental HT29 and HCT116 cells and further validated by examining the IC50 with 5-Fu treatment (Fig. 6A). Moreover, the results further showed that the chemoresistant HCT116R and HT29R cells exhibited a stronger cell viability and colony formation capabilities compared to the parental cells (Fig. 6B, C). Likewise, the xenograft model indicated that chemoresistant increased the efficacy of in vivo tumor development (Fig. 6D–G). The IB results suggested that survivin was increased in 5-Fu-resistance CRC cells (Fig. 6H). To determine whether 5-Fu-resistance was the result of survivin upregulation in CRC cells, we constructed HCT116R and HT29R cells with survivin knockdown (Fig. 6I). The results discovered that 5-Fu decreased cell viability, colony formation, and increased cleaved-caspase 3 protein levels in HCT116R and HT29R cells with survivin knockdown (Fig. 6J–L). These results revealed that resistance to 5-Fu depends on survivin expression in CRC cells.

### Tan IIA overcomes chemoresistant in CRC cells

**Fig. 7:**
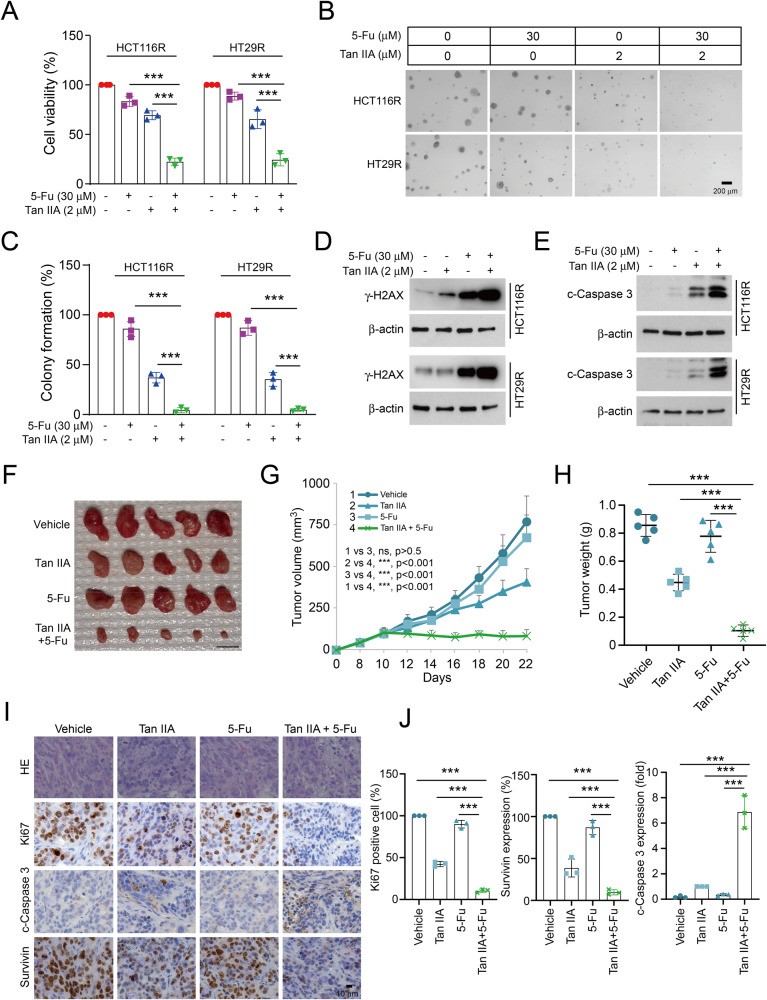
Tan IIA overcomes 5-Fu resistance in CRC cells. **A**–**C** HCT116R and HT29R cells were treated with 5-Fu (30 μM), Tan IIA (2 μM), or in combination. Cell viability and colony formation were detected using MTS (**A**) and soft agar assay (**B** representative image; **C** qualification). IB assay of γ-H2AX (**D**) and cleaved-caspase 3 (**E**) in HCT116R and HT29R cells with 5-Fu (30 μM), tanshinone IIA (2 μM) or combination treatment. The tumor mass (**F**), volume (**G**) and weight (**H**) of HT29R tumors with various treatments. Scale bar, 1 cm. **I** Ki67, cleaved-caspase 3 and survivin were analyzed by IHC staining in HT29R xenograft tumors. Scale bar, 25 μm. **J** Qualification of IHC staining of Ki67, cleaved-caspase 3 and survivin. ***P < 0.001.

To determine the inhibitory effect of Tan IIA on chemoresistant CRC cells (HCT116R and HT29R), we performed MTS assay to detect the viability of CRC cells. The results suggested that Tan IIA, but not 5-Fu, diminished cell viability significantly in HCT116R and HT29R cells (Fig. 7A). Combination with Tan IIA increased the inhibitory efficacy of 5-Fu on chemoresistant CRC cells (Fig. 7A), which was further confirmed by soft agar assay (Fig. 7B, C). Moreover, the immunoblotting results suggested that the protein levels of γ-H2AX (Fig. 7D), the DNA-damage marker, were upregulated in the combination-treated groups, indicating that Tan IIA augmented 5-Fu-induced DNA damage in chemoresistant cells. Tan IIA alone, or the combination of Tan IIA with 5-Fu, promoted the expression of cleaved-caspase 3 (Fig. 7E), suggesting that Tan IIA resensitized HCT116R and HT29R cells to 5-Fu treatment. We next established the in vivo xenograft mouse model using the HT29R cells and found that the tumor mass (Fig. 7F), growth curve (Fig. 7G), and weight (Fig. 7H) were suppressed significantly after Tan IIA or combination treatment. However, 5-Fu alone failed to do so. Furthermore, the IHC data showed that combining Tan IIA with 5-Fu significantly decreased Ki67 and survivin protein levels in HT29R-derived tumors compared to that of 5-Fu alone or vehicle-treated tumors (Fig. 7I, J). These results revealed that Tan IIA overcomes chemoresistant to 5-Fu in CRC cells.

## Discussion

Irinotecan or oxaliplatin combined with leucovorin and 5-Fu is the first-line treatment in advanced colorectal cancer [6]. However, primary or acquired chemoresistance during chemotherapy remains challenging. Numerous studies have revealed that tumor cell heterotrophy, cancer stem cells, changes in chemotherapeutic agents’ targeted sites, and tumor apoptosis inhibition are responsible for chemotherapy resistance in CRC [32]. Liu et al. found that protein tyrosine kinase 6 was overexpressed in CRC tissues and associated with chemoresistance via activating JAK2/STAT3 signaling to promote the stemness of CRC cells [33]. A serine/threonine kinase with a U2AF homology motif, UHMK1, was observed that was frequently upregulated in CRC samples and promoted cell proliferation and oxaliplatin resistance by augmenting IL-6/STAT3 signaling [34]. In this present study, our findings indicated that survivin expression increased in 5-Fu resistant cell lines, while depletion of survivin significantly re-sensitized CRC cells to 5-Fu treatment.

Survivin, encoded by the gene Baculoviral IAP Repeat Containing 5, is frequently overexpressed in tumor tissues and is considered an unfavorable prognostic marker due to its role in the inhibition of apoptosis, lymph node invasion, metastasis, tumor angiogenesis, stemness and chemoresistance [35, 36]. The regulation of survivin expression and function is intricated and present at different levels, including transcription, differential splicing, intracellular sequestration via different ligands, post-translational modification, and protein degradation [10, 31]. Several signaling pathways and related molecules include PI3K/AKT, JAK/STAT, NF-κB, TGF-β, WNT/β-Catenin, NOTCH, and SMAD, and have been verified to be involved in survivin expression dysregulation in cancer cells [37, 38]. The mitotic kinases polo-like kinase 1 and Aurora kinase B, as survivin activating kinases, were overexpressed in (TNBC) and significantly affected cell proliferation and cell cycle progression in African Americans TNBC cells via increasing survivin phosphorylation [39]. Chang et al. identified that oncogenic KRAS upregulated survivin expression by activating extracellular-signal-regulated kinase 1/2 (ERK1/2) in pancreatic cancer cells, which in turn protected MYC from degradation to promote malignant transformation [40].

With the gradual clarification of the biological function and regulatory mechanism of Survivin, increasing novel therapeutic strategies are devoted to downregulating survivin expression and even expect to increase the sensitivity of cancer cells to antitumor drugs. Currently, utilizing the RNA interfering mechanism, antisense oligonucleotides, ribozymes, small molecule inhibitors, cancer vaccines, etc., have been developed to reduce survivin expression [17, 37, 41]. (-)-gossypol, a male contraceptive, sensitized hepatocellular carcinoma cells to epirubicin (EPI) via attenuating EPI-induced upregulation of survivin [42]. A novel survivin dimerization inhibitor LQZ-7F1 was synthesized, which could effectively induce survivin degradation and exhibit a strong synergistic effect with docetaxel in inhibiting prostate cancer [43]. Additionally, the natural product xanthohumol was unveiled to inhibit the tumorigenic properties of oral squamous cell carcinoma cells via reducing survivin phosphorylation at Thr34 and facilitating ubiquitination, thus resulting in survivin degradation [44]. In the present study, administration of Tan IIA significantly inhibited proliferation and activated apoptosis of CRC cells by downregulating survivin. Mechanistic studies indicated that the Akt/Wee1/CDK1 signaling pathway was suppressed followed by Tan IIA treatment, resulting in the downregulation of survivin phosphorylation at Thr34 and destruction of the interaction between survivin and USP1, ultimately inducing survivin degradation. Moreover, the combination of Tan IIA and 5-Fu treatment markedly restored the antitumor effect of 5-Fu on chemoresistant CRC cells.

In summary, the present study demonstrated that the natural compound Tan IIA exerted strong antitumor activity in CRC cells. Tan IIA significantly inhibited cell proliferation, induced apoptosis activation, and re-sensitized resistant cells to 5-Fu treatment by disrupting the protective effect of USP1 on its protein stability. This study suggested that Tan IIA may act as an effective antitumor agent to induce survivin degradation in CRC cells, which provided a novel avenue for the combination of survivin inhibitors and traditional chemotherapeutic agents to overcome chemoresistance with aims to improve therapeutic outcomes in patients with CRC.

## Materials and methods

### Cell culture and reagents

Human CRC cell lines HCT116, HT29, and HCT8 were purchased from American Type Culture Collection (ATCC, Manassas, VA). All cells were cultured in a humidified incubator at 37°C with 5% CO2 and subjected to mycoplasma analysis every 2 months. RPMI-1640 and DMEN cell culture media, and Fetal Bovine Serum (FBS) were obtained from Invitrogen (Grand Island, NY). Transfection reagent lipofectamine 2000 was purchased from Thermo Fisher Scientific (Waltham, MA). The natural products Tan IIA, MG132, cycloheximide (CHX), and 5-Fu were obtained from Selleck Chemicals (Houston, TX). SDS, DMSO, NaCl, and Trisbase for buffer preparation were purchased from Sigma-Aldrich (Merck KGaA; St. Louis, MO, USA). Antibodies against survivin (#2808), cleaved-caspase 3 (#9664), Bax (#5023), VDAC1 (#4661), cytochrome C (#11940), ubiquitin (#3936), p-Akt Ser473 (#4060), p-CDK1 Thr161 (#9114), p-Wee1 Ser642 (#4910), p-survivin Thr34 (#8888), USP1 (#8033), Flag-tag (#8146), α-Tubulin (#3873) and β-actin (#4970) were purchased from Cell Signaling Technology, Inc. (Beverly, MA). Ki67 (#ab16667) antibodies were obtained from Abcam (Cambridge, United Kingdom).

### MTS assay

To determine cell viability, MTS assay was performed as described previously [45]. Briefly, CRC cells were seeded into the 96-well plates (2 × 10^3^ cells/well) and maintained overnight, followed by incubation with different concentrations of Tan IIA at various times. Then MTS reagent (#G3580, Madison, WI) was added to the cell culture medium, and cell viability was detected according to the standard protocol.

### Western blotting

CRC cells administrated with Tan IIA were lysed in RIRA buffer (#89901, Thermo Fisher Scientific) at 4°C for 30 min. Whole-cell extract (WCE) was collected after centrifugation at 12,000 rpm for 10 min, and the protein concentration was detected using the BCA protein assay kit (#23228, Thermo Fisher Scientific. WCE was then subjected to SDS-PAGE gels and transferred to the PVDF membrane. Subsequently, the membranes were blocked with 5% non-fat milk for 1 h and incubated with the primary and second antibodies. Finally, the expression of the target was visualized using the ECL reagent (#34579, Thermo Fisher Scientific).

### Anchorage-independent cell growth

The Anchorage-independent cell growth assay was performed as described previously [23]. Eagle’s basal medium containing 10% FBS, 0.6% agar, and various concentration of Tan IIA were loaded in a 6-well plate as an agar base. CRC cells were resuspended and inoculated in 6-well plates (8 × 10^3^ cells/well) with 0.6% Basal Medium Eagle (0.3% agar, 10% FBS, and different concentrations of Tan IIA). After maintaining for 2 weeks, the colonies were counted.

### Generation of survivin knockdown stable cell lines

The pLKO.1-shSurvivin lentivirus plasmids (TRCN0000073718, TRCN0000073721, Millipore Sigma), PSPAX2, and PMD2-G were co-transfected into 293 T cells to construct survivin knockdown stable cells. After transfection 72 h, the supernatant containing viral was collected. The lentivirus and polybrene (5 µg/ml) were added when CRC cells were grown at 70–80% confluence. The successfully infected cells were screened using cell culture medium containing puromycin (1 μg/ml) for 2 weeks.

### Co-immunoprecipitation (Co-IP)

The cell lysates were prepared with the IP buffer (#87787, Thermo Fisher Scientific) and then used for the Co-IP assay. Briefly, cell lysate incubated with primary antibodies against survivin or USP1 overnight at 4°C with rotation. Protein A/G agarose beads were added to each cell lysate and incubated with mild rotation at 4 °C for 2 h. The agarose beads were then washed with PBS and cell lysis buffer. Finally, 1xSDS-PAGE loading buffer was used to resuspend the agarose beads, then boiled for 5 min and centrifuged at 12,000 rpm for 10 min to collect the supernatant for western blot analysis.

### In vivo tumor growth

The in vivo tumorigenesis was approved by the Institutional Animal Care and Use Committee of the Third Xiangya Hospital of Central South University (Changsha, China). The parental or 5-Fu-resistant CRC cells (2 × 10^6^) were injected into the right flank of 6-week-old athymic nude mice (*n* = 5). Tumor volume was monitored and measured every 2 days. When tumor volume reached around 100 mm3, the tumor-bearing mice were randomly divided into three groups (*n* = 4). The treatment group was administrated Tan IIA (Low: 5 mg/kg every 2 days; High: 15 mg/kg every 2 days), and the control group initiated the vehicle control via intraperitoneal injection. For combined treatment, the tumor-bearing mice were randomly divided into four groups (*n* = 5): 1, vehicle control (0.5% dimethyl sulfoxide in 100 µL Corn oil /every 2 days, i.p.); 2, Tan IIA (5 mg/kg/ in 100 µL Corn oil every 2 days, i.p.); 3, 5-Fu (4 mg/kg/ in 100 µL Corn oil every 4 days, i.p.); 4, Tan IIA (5 mg/kg/ every 2 days, i.p.) + 5-Fu (4 mg/kg/ every 4 days, i.p.). Finally, the mice were euthanized with CO_2_ (3 L/min) for 5 min to collect tumor tissues. Tumor volume was calculated following the formula: length × width × width/2.

### Immunohistochemical staining (IHC)

The IHC staining was performed as described previously [46]. Tumor tissues obtained from xenograft tumors were analyzed using IHC. Briefly, the tissue sections were deparaffinized and rehydrated, immersing in sodium citrate buffer (10 mM, pH 6.0) subsequently and boiling for 10 min to repair the antigen. The tissue slides were treated with 3% H2O2 in methanol for 10 min, washed with PBS, and blocked with 10% goat serum albumin, and then incubated with primary antibodies overnight at 4°C. The target protein was visualized using the DAB substrate and counterstained by hematoxylin following hybridization with secondary antibodies at room temperature.

### Statistical analysis

Data were performed from at least three independent determinations and represented as mean ± sd. GraphPad Prism 5 (GraphPad 5.0, San Diego, CA, USA) software was used for statistical analysis. Statistical comparisons between different groups were analyzed by Student’s t-test or ANOVA. A probability value of *p* < 0.05 was used as the criterion for statistical significance.

### Supplementary information

WB full gel

aj-checklist

## Data Availability

The datasets generated during and/or analysed during the current study are available from the corresponding author on reasonable request.

## References

[1] Sung H, Ferlay J, Siegel RL, Laversanne M, Soerjomataram I, Jemal A (2021). Global cancer statistics 2020: GLOBOCAN estimates of incidence and mortality worldwide for 36 cancers in 185 countries. CA Cancer J Clin.

[2] Li N, Lu B, Luo C, Cai J, Lu M, Zhang Y (2021). Incidence, mortality, survival, risk factor and screening of colorectal cancer: a comparison among China, Europe, and northern America. Cancer Lett.

[3] Rawla P, Sunkara T, Barsouk A (2019). Epidemiology of colorectal cancer: incidence, mortality, survival, and risk factors. Prz Gastroenterol.

[4] Benson AB, Venook AP, Al-Hawary MM, Cederquist L, Chen Y-J, Ciombor KK (2018). Anal Carcinoma, Version 2.2018, NCCN clinical practice guidelines in oncology. J Natl Compr Canc Netw.

[5] Weng J, Zhang Y, Liang W, Xie Y, Wang K, Xu Q (2023). Downregulation of CEMIP enhances radiosensitivity by promoting DNA damage and apoptosis in colorectal cancer. Med Oncol.

[6] McQuade RM, Stojanovska V, Bornstein JC, Nurgali K (2017). Colorectal cancer chemotherapy: the evolution of treatment and new approaches. Curr Med Chem.

[7] Singh P, Lim B (2022). Targeting apoptosis in cancer. Curr Oncol Rep.

[8] Morana O, Wood W, Gregory CD (2022). The apoptosis paradox in cancer. Int J Mol Sci.

[9] Warren CFA, Wong-Brown MW, Bowden NA (2019). BCL-2 family isoforms in apoptosis and cancer. Cell Death Dis.

[10] Mita AC, Mita MM, Nawrocki ST, Giles FJ (2008). Survivin: key regulator of mitosis and apoptosis and novel target for cancer therapeutics. Clin Cancer Res.

[11] Altieri DC (2003). Survivin, versatile modulation of cell division and apoptosis in cancer. Oncogene..

[12] Altieri DC (2001). The molecular basis and potential role of survivin in cancer diagnosis and therapy. Trends Mol Med.

[13] LaCasse EC, Baird S, Korneluk RG, MacKenzie AE (1998). The inhibitors of apoptosis (IAPs) and their emerging role in cancer. Oncogene..

[14] Zhang Y, Sun Y, Jia Y, Zhang Q, Zhu P, Ma X (2021). α5-nAChR and survivin: Two potential biological targets in lung adenocarcinoma. J Cell Physiol.

[15] Martínez-Sifuentes MA, Bassol-Mayagoitia S, Nava-Hernández MP, Ruiz-Flores P, Ramos-Treviño J, Haro-Santa Cruz J (2022). Survivin in breast cancer: a review. Genet Test Mol Biomark.

[16] Chen W-C, Liu Q, Fu J-X, Kang S-Y (2004). Expression of survivin and its significance in colorectal cancer. World J Gastroenterol.

[17] Warrier NM, Krishnan RK, Prabhu V, Hariharapura RC, Agarwal P, Kumar P (2022). Survivin inhibition by piperine sensitizes glioblastoma cancer stem cells and leads to better drug response. Int J Mol Sci.

[18] Fäldt Beding A, Larsson P, Helou K, Einbeigi Z, Parris TZ (2022). Pan-cancer analysis identifies BIRC5 as a prognostic biomarker. BMC Cancer.

[19] Dong X, Liu W, Li X, Gan Y, Zhou L, Li W (2022). Butein promotes ubiquitination-mediated survivin degradation inhibits tumor growth and overcomes chemoresistance. Sci Rep.

[20] Zhang Y, Liu K, Yan C, Yin Y, He S, Qiu L (2022). Natural polyphenols for treatment of colorectal cancer. Molecules..

[21] Huang X-M, Yang Z-J, Xie Q, Zhang Z-K, Zhang H, Ma J-Y (2019). Natural products for treating colorectal cancer: a mechanistic review. Biomed Pharmacother.

[22] Zhong C, Lin Z, Ke L, Shi P, Li S, Huang L (2021). Recent research progress (2015-2021) and perspectives on the pharmacological effects and mechanisms of tanshinone IIA. Front Pharm.

[23] Li M, Liu H, Zhao Q, Han S, Zhou L, Liu W (2021). Targeting Aurora B kinase with Tanshinone IIA suppresses tumor growth and overcomes radioresistance. Cell Death Dis.

[24] Fang Z-Y, Zhang M, Liu J-N, Zhao X, Zhang Y-Q, Fang L (2020). Tanshinone IIA: a review of its anticancer effects. Front Pharm.

[25] Khan FB, Singh P, Jamous YF, Ali SA, Abdullah, Uddin S (2022). Multifaceted pharmacological potentials of curcumin, genistein, and tanshinone iia through proteomic approaches: an in-depth review. Cancers.

[26] Li M, Gao F, Zhao Q, Zuo H, Liu W, Li W (2020). Tanshinone IIA inhibits oral squamous cell carcinoma via reducing Akt-c-Myc signaling-mediated aerobic glycolysis. Cell Death Dis.

[27] Li H, Wu M, Guo C, Zhai R, Chen J (2022). Tanshinone IIA regulates keap1/Nrf2 signal pathway by activating sestrin2 to restrain pulmonary fibrosis. Am J Chin Med.

[28] Xu L, He D, Wu Y, Shen L, Wang Y, Xu Y (2022). Tanshinone IIA inhibits cardiomyocyte apoptosis and rescues cardiac function during doxorubicin-induced cardiotoxicity by activating the DAXX/MEK/ERK1/2 pathway. Phytomedicine..

[29] Liu J, Zhang C, Liu S, Wang X, Wu X, Hao J (2022). Tanshinone IIA promotes apoptosis by downregulating BCL2 and upregulating TP53 in triple-negative breast cancer. Naunyn Schmiedebergs Arch Pharmacol..

[30] Gao F, Li M, Liu W, Li W (2020). Inhibition of EGFR signaling and activation of mitochondrial apoptosis contribute to Tanshinone IIA-mediated tumor suppression in non-small cell lung cancer cells. Onco Targets Ther.

[31] Woo SM, Kim S, Seo SU, Kim S, Park J-W, Kim G (2022). Inhibition of USP1 enhances anticancer drugs-induced cancer cell death through downregulation of survivin and miR-216a-5p-mediated upregulation of DR5. Cell Death Dis.

[32] Chen L, Yang F, Chen S, Tai J (2022). Mechanisms on chemotherapy resistance of colorectal cancer stem cells and research progress of reverse transformation: A mini-review. Front Med.

[33] Liu C, Pan Z, Chen Q, Chen Z, Liu W, Wu L (2021). Pharmacological targeting PTK6 inhibits the JAK2/STAT3 sustained stemness and reverses chemoresistance of colorectal cancer. J Exp Clin Cancer Res.

[34] Gao X, Bao W, Bai J, Fan K, Li L, Li Y (2022). UHMK1 aids colorectal cancer cell proliferation and chemoresistance through augmenting IL-6/STAT3 signaling. Cell Death Dis.

[35] Wheatley SP, Altieri DC (2019). Survivin at a glance. J Cell Sci.

[36] Chen Y, Ye B, Wang C, Nie Y, Qin J, Shen Z (2022). PLOD3 contributes to HER-2 therapy resistance in gastric cancer through FoxO3/Survivin pathway. Cell Death Discov.

[37] Warrier NM, Agarwal P, Kumar P (2020). Emerging importance of survivin in stem cells and cancer: the development of new cancer therapeutics. Stem Cell Rev Rep.

[38] Wang H, Tan Y, Jia H, Liu D, Liu R (2022). Posaconazole inhibits the stemness of cancer stem-like cells by inducing autophagy and suppressing the Wnt/β-catenin/survivin signaling pathway in glioblastoma. Front Pharm.

[39] Garlapati C, Joshi S, Bhattarai S, Krishnamurthy J, Turaga RC, Nguyen T (2023). PLK1 and AURKB phosphorylate survivin differentially to affect proliferation in racially distinct triple-negative breast cancer. Cell Death Dis.

[40] Chang W-H, Liu Y, Hammes EA, Bryant KL, Cerione RA, Antonyak MA (2022). Oncogenic RAS promotes MYC protein stability by upregulating the expression of the inhibitor of apoptosis protein (IAP) family member Survivin. J Biol Chem.

[41] Shojaei F, Yazdani-Nafchi F, Banitalebi-Dehkordi M, Chehelgerdi M, Khorramian-Ghahfarokhi M (2019). Trace of survivin in cancer. Eur J Cancer Prev.

[42] Jiang W, Wang W, Sun L, Xiao Y, Ma T, Li B (2022). (-)-Gossypol enhances the anticancer activity of epirubicin via downregulating survivin in hepatocellular carcinoma. Chem Biol Interact.

[43] Peery R, Cui Q, Kyei-Baffour K, Josephraj S, Huang C, Dong Z (2022). A novel survivin dimerization inhibitor without a labile hydrazone linker induces spontaneous apoptosis and synergizes with docetaxel in prostate cancer cells. Bioorg Med Chem.

[44] Li M, Gao F, Yu X, Zhao Q, Zhou L, Liu W (2020). Promotion of ubiquitination-dependent survivin destruction contributes to xanthohumol-mediated tumor suppression and overcomes radioresistance in human oral squamous cell carcinoma. J Exp Clin Cancer Res.

[45] Li W, Yu X, Xia Z, Yu X, Xie L, Ma X (2018). Repression of Noxa by Bmi1 contributes to deguelin-induced apoptosis in non-small cell lung cancer cells. J Cell Mol Med.

[46] Yu X, Wang R, Zhang Y, Zhou L, Wang W, Liu H (2019). Skp2-mediated ubiquitination and mitochondrial localization of Akt drive tumor growth and chemoresistance to cisplatin. Oncogene..

