# Supplementary material for: Targeting survivin with Tanshinone IIA inhibits tumor growth and overcomes chemoresistance in colorectal cancer
Source: Cell Death Discov. 2023 Sep 25;9:351. doi: 10.1038/s41420-023-01622-8 (PMC10520088; doi:10.1038/s41420-023-01622-8)

Full gel for Figure 1

E

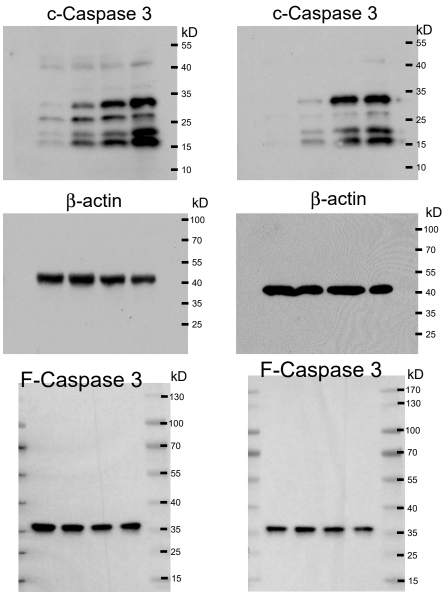

F

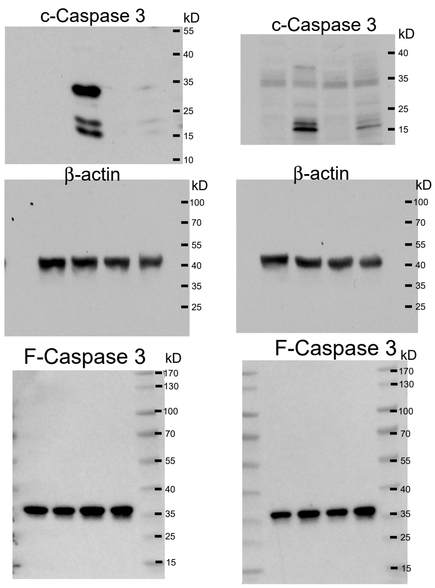

H

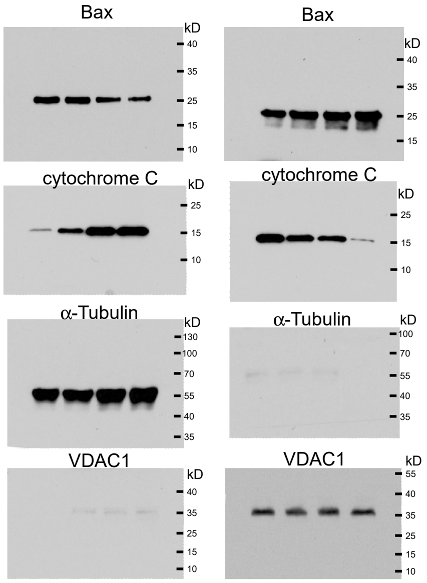

Full gel for Figure 2

A

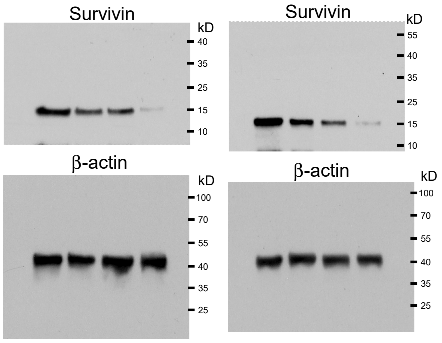

B

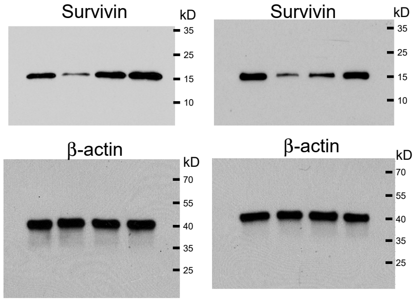

C

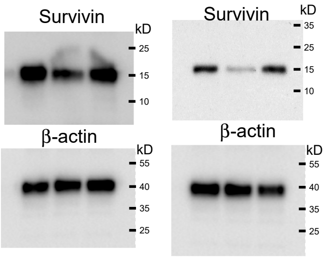

D

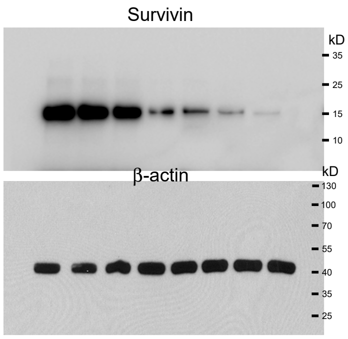

E

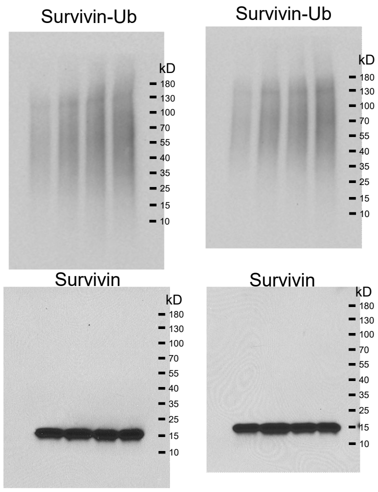

F

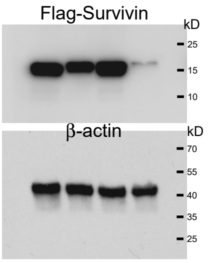

Full gel for Figure 3

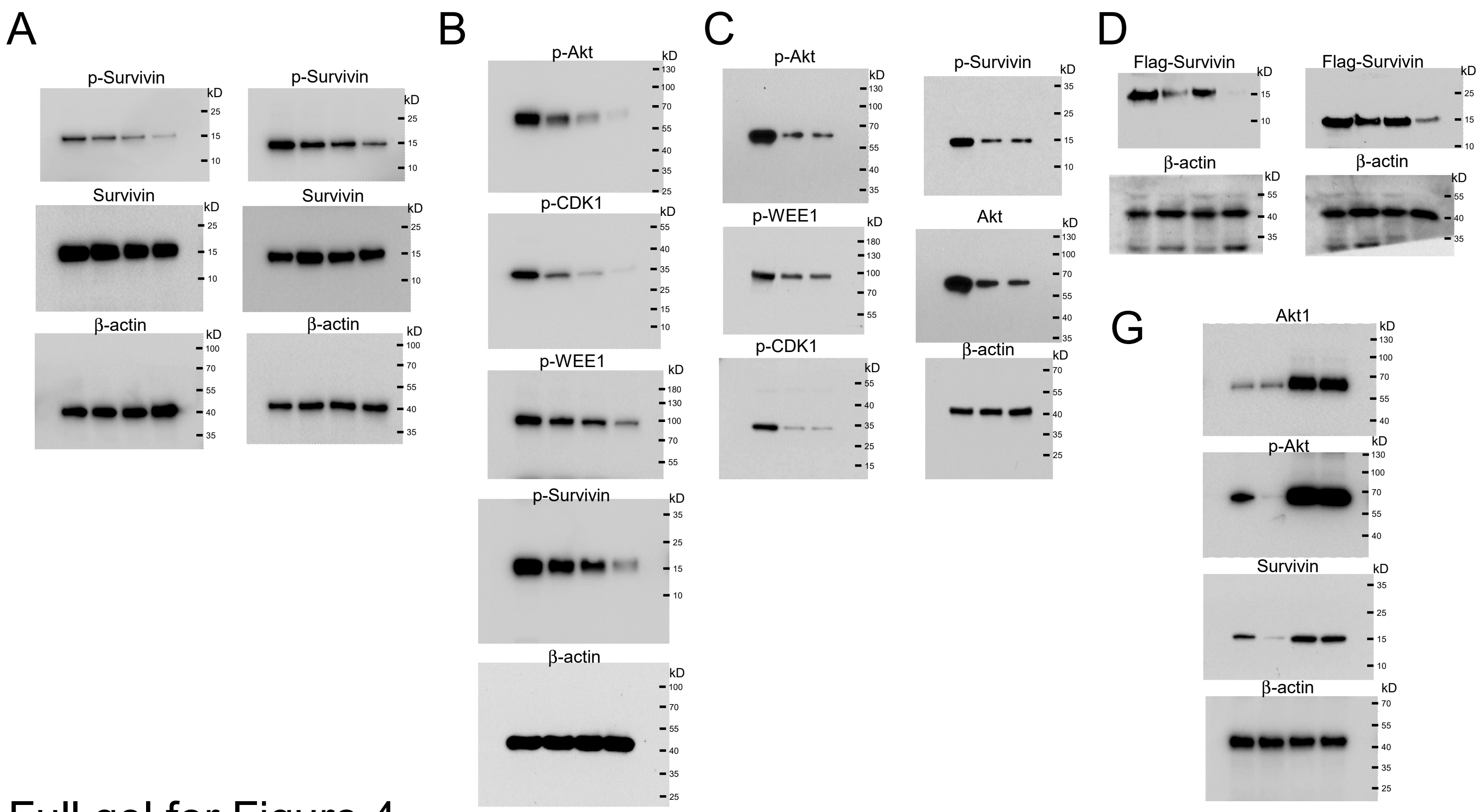

Full gel for Figure 4

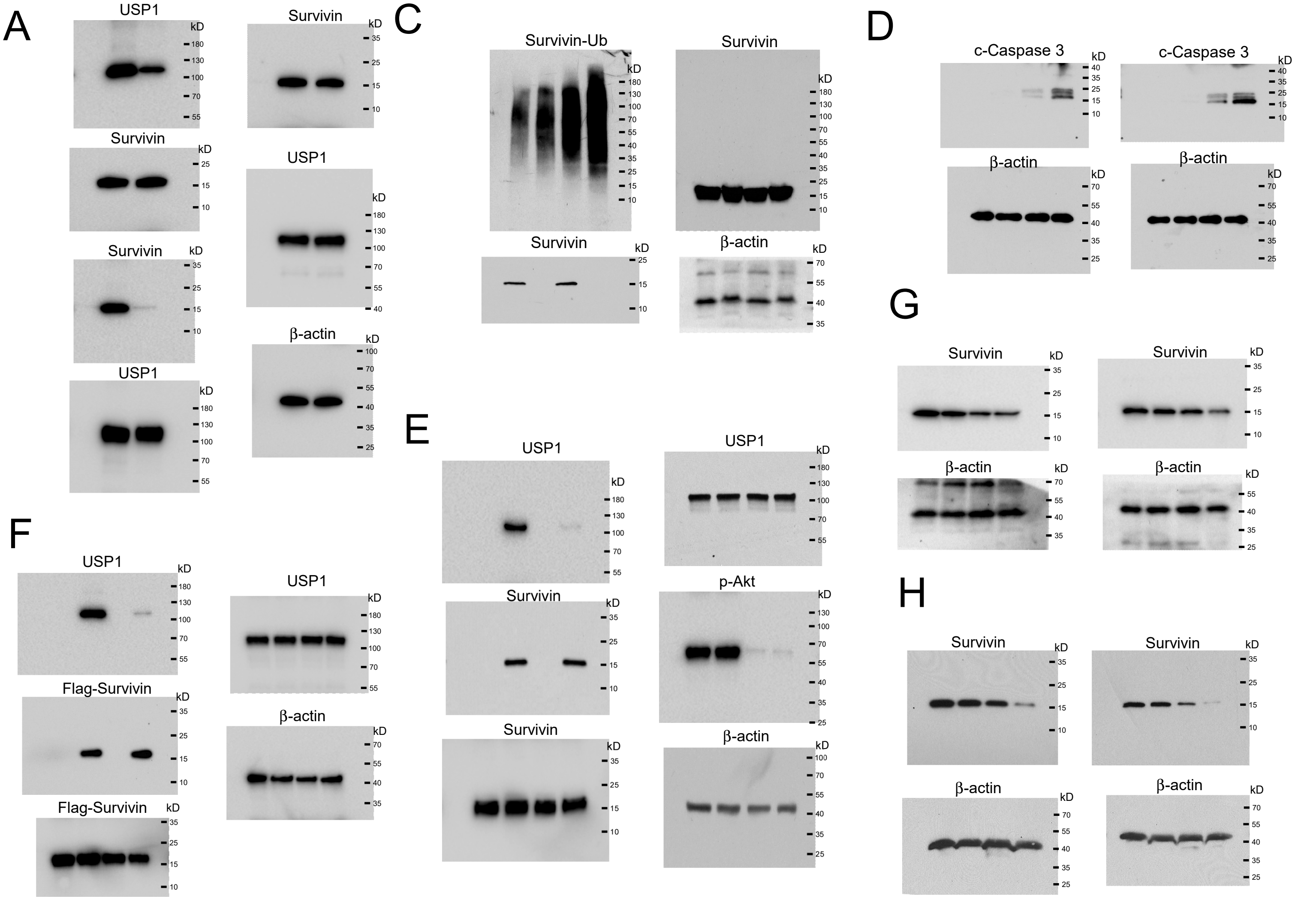

Full gel for Figure 6

H

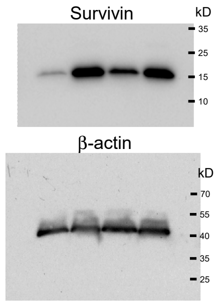

I

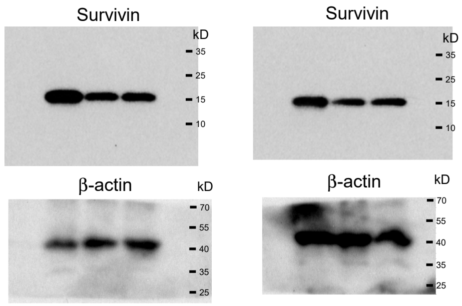

L

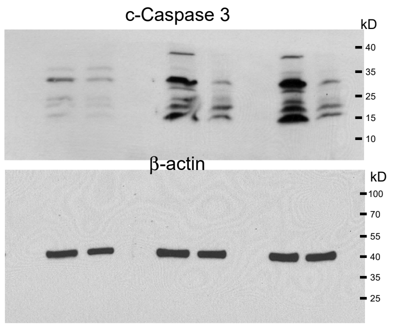

Full gel for Figure 7

D

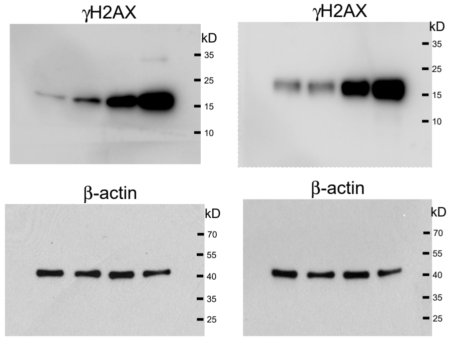

E

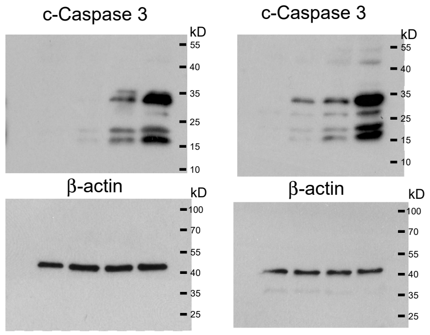

Supplement: Supplementary file 1 — WB full gel [file 41420_2023_1622_MOESM1_ESM.pdf]
